# Undertesting of COVID-19 in Indonesia: what has gone wrong?

**DOI:** 10.7189/jogh.10.020306

**Published:** 2020-12

**Authors:** Giovanni van Empel, Joko Mulyanto, Bayu Satria Wiratama

**Affiliations:** 1 Department of Health Policy and Management, Faculty of Medicine, Public Health and Nursing, Universitas Gadjah Mada, Yogyakarta, Indonesia; 2 Centre for Health Economics, Monash Business School, Monash University, Melbourne, Australia; 3 Department of Public Health and Community Medicine, Faculty of Medicine, Universitas Jenderal Soedirman, Purwokerto, Indonesia; 4 Department of Public and Occupational Health, Amsterdam University Medical Centre, University of Amsterdam, Amsterdam Public Health research institute, Amsterdam, the Netherlands; 5 Department of Biostatistics, Epidemiology and Population Health, Faculty of Medicine, Public Health and Nursing, Universitas Gadjah Mada, Yogyakarta, Indonesia; 6 Graduate Institute of Injury Prevention and Control, College of Public Health, Taipei Medical University, Taipei, Taiwan

Since reporting its first COVID-19 case on March 2nd, as of April 29 Indonesia has tested 67 784 suspected cases, a mere 0.02% of the total population [1]. Over the span of nearly eight weeks from the first reported case, that equates to 247 tests per million residents (see **Figure 1**). This places Indonesia at the second lowest among South East Asian countries. To draw a comparison, Singapore and Malaysia have nearly 20 000 and 4700 tests per million residents, respectively.

**Figure 1:**
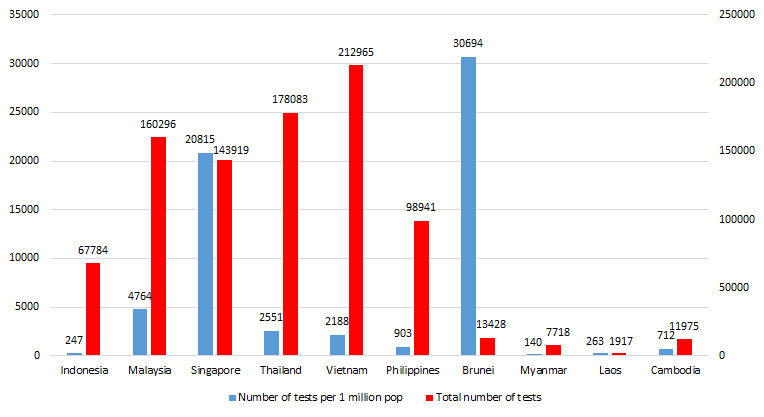
Comparison of number test per 1 million populations with neighbouring countries (per April 29, 2020). Data source: WorldOMeters [2].

A country of 267 million and the fourth largest in the world, Indonesia reports a relatively small number of confirmed COVID-19 cases compared to other affected countries. Until 29 April 2020, official data showed that Indonesia had 9771 confirmed cases and 784 confirmed deaths [1]. With a case fatality rate of 8.0%, this puts Indonesia at the top among neighbouring countries, and 9th globally [2]. This raises a lot of questions surrounding the general capacity of COVID-19 testing in Indonesia.

Many experts believe that the low number of cases were massively underreported due to our low testing capacity. This concern is substantiated by the low number of tests by the Indonesian health authority. Moreover, throughout the first month following the first reported case, Indonesia had only averaged around 27 tests per day.

## QUESTIONABLE POLICY RESPONSE

Reports of downplaying the risk of COVID-19 by Indonesian authorities is well documented [3]. Weeks before the first reported case, on one occasion at a Parliament hearing, the Indonesian Health Minister said that strategies and measures to prevent COVID-19 are in place [4]. The reality seems to be the opposite.

The first official testing protocol for COVID-19 was published on March 16, just two weeks after the Ministry of Health reported the first case. The protocol requires all specimens to be examined at the Central Laboratory of the National Institute of Health Research and Development, Ministry of Health located in the capital Jakarta. This means hospitals throughout Indonesia were required to send the specimen to Jakarta, creating significant delays – up to 10 days – for the results to be finalised.

This requirement also created a massive bottleneck of the testing process due to mismatches in the resource capacity and the demand for high volume testing. Not long after the first protocol was published, the Ministry of Health (MoH) expanded designated test centres to ten MoH owned laboratories across Indonesia. While this move looks like a proper response, there were questions on the non-involvement of non MoH owned experienced laboratory centres available within the Indonesian Medical Schools. Research suggests that health laboratory infrastructure is one weak link in the health system [5].

The reluctance by the Health authority to decentralise the testing process persists amid enormous public pressure and even when provincial leaders have shown initiative in assisting the testing process. Only after the President formally instructed to expand the testing capacity in the early April 2020 (4 weeks after the initial confirmed case was recorded), then the health authority started to decentralise the testing process to 48 public-owned laboratories. The Government has set an ambitious target to achieve 10 000 tests per day in immediate time albeit no clear timeline for the target. While the number of RT-PCR machines are relatively sufficient, there is a shortage of the testing kits such as reagent and Virus Transport Media which likely need to import from outside Indonesia. These shortages are foreboding with the demand for tests increasing to around 2000 tests per day (a number which is yet still far below the aimed target).

**Figure:**
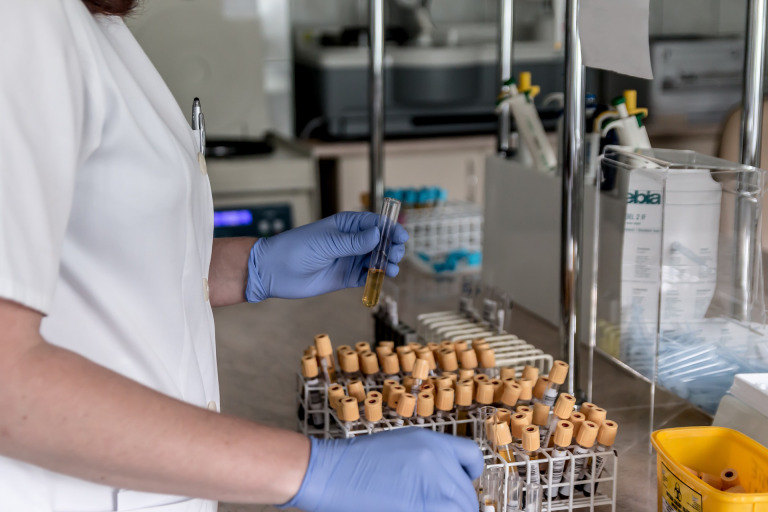
Photo: Image by Michal Jarmoluk from Pixabay.

Testing protocol is also a contributing factor to the low number of testing. Contrary to WHO guidelines, the official national guidelines [6] distinguishes suspect cases into two different categories. The first category is called person under surveillance which is defined as individuals who have mild acute respiratory infection symptoms with travel history from outbreak area or history of close contact to confirmed cases. Per April 29, there are more than 200 000 cases identified in this category. The second category is patients under surveillance which defined as individuals who have moderate or severe acute respiratory infection such as pneumonia with similar travel history or close contact. As of April 29, almost 21 000 people categorized as patients under surveillance. Only individuals in the latter category are eligible to be tested. This very strict criteria limit the number eligible of testing. The guidelines have subsequently led to a failure in testing suspected cases who only have mild symptoms or asymptomatic.

## MOVING FORWARD

There is a need for fundamental policy changes to adequately address the COVID-19 pandemic in Indonesia. First, we need to provide a stronger stewardship to contain COVID-19 through a more concerted effort. This means continuously expanding the testing capacity by involving private laboratories. Strategically encouraging domestic production of the testing kit may be a sustainable solution. This means accurately identifying the real capacity parallel with ensuring efficient distribution of tests and testing resources. There are other areas of improvement as well. Publication of genomic sequencing, for instance, could provide better understanding of the type of virus Indonesia is facing. Lastly maintaining the broader testing criteria introduced in the latest version of national protocol and effectively collaborating with all stakeholders will be key to survive this pandemic wave.
